# Prevalence of Blood Transfusion and Factors Influencing Blood Loss Following Primary Total Knee Replacement Surgery

**DOI:** 10.5704/MOJ.2503.007

**Published:** 2025-03

**Authors:** AN Sadagatullah, MAA Sahadun, MK Md-Isa, MF Yusof

**Affiliations:** 1 Department of Orthopaedics, Universiti Sains Malaysia, Kubang Kerian, Malaysia; 2 Department of Orthopaedics, Hospital Melaka, Melaka, Malaysia

**Keywords:** total knee replacement (TKR), allogenic blood transfusion (ABT), blood loss in surgery

## Abstract

**Introduction:**

Total knee replacement (TKR) is a highly effective treatment for end-stage knee osteoarthritis and has been proven to have excellent results in relieving pain as well as improving mobility of the patient. Although becoming more increasingly performed, it is still associated with considerable perioperative blood loss requiring allogenic blood transfusion. Allogenic blood transfusion (ABT) can be lifesaving in certain clinical situations but also comes with their own risks and side effects. The reported incidence of ABT and blood loss following TKR surgery varies widely in the literature. The objectives of this study were to look at the prevalence of ABT, factors leading to transfusion as well as increase in blood loss.

**Material and Methods:**

A cross-sectional retrospective study was conducted involving 296 adult patients who underwent elective primary unilateral TKR surgery from January 2015 until December 2019 at Hospital Melaka. Medical records of these patients were reviewed, and relevant data were extracted for final analysis. Incidence of ABT, demographic data, use of antiplatelet/anticoagulant, tourniquet time, types of general anaesthesia, and pre- and post-operative haemoglobin count were recorded. These factors were analysed to look at the association with ABT as well as increase in blood loss.

**Results:**

Prevalence of ABT following primary unilateral TKR surgery were found to be 4.39% (95% confidence interval 2.04, 6.74). Pre-operative haemoglobin value was found to be the only significant variable associated with blood transfusion [P<0.001; Odds ratio (OR) = 0.35; 95% Confidence interval (CI) 0.22, 0.54]. Meanwhile, prolonged tourniquet time of >120 minutes was the only significant variable towards an increase in blood loss. Participants with tourniquet time >120 minutes has 2.67 times the odds to have blood loss >2 g/dL compared to participants with tourniquet time of less or equal to 120 minutes (95% CI=1.54, 4.64).

**Conclusion:**

The prevalence of ABT following primary unilateral TKR was lower in our centre compared to other reported studies. Pre-operative optimisation of anaemic patients with haematinics will help surgeons reduce the need for ABT.

## Introduction

Total knee replacement (TKR) is a highly effective treatment for end-stage knee arthritis. This procedure has been proven to have excellent results in relieving pain as well as improving mobility of the patient^1^. In the U.S., demand for this procedure is expected to grow following an increase in life expectancy and medical advancement. In 2010, the CDC estimated that 719,000 patients underwent total knee replacement in the U.S. This number is projected to reach 3.5 million annually by the year 20302.

Although becoming more increasingly performed, TKR is still associated with considerable perioperative blood loss and blood transfusion which have been attributed to extensive soft tissue release and bone cuts^3^. Multiple techniques have been described to minimise intra-operative blood loss, which includes the use of a torniquet^4^, utilisation of computer-assisted surgery^5^, sealing of the femoral intramedullary canal^6^, and the use of antifibrinolytic agents^7^. However, different arguments on the efficacy of these blood-conserving strategies have been reported in the literature.

While the use of tourniquet has been justified as a good surgical practice and significantly reduces intra-operative blood loss, it does not help in reducing post-operative blood loss. The opponents of the use of a tourniquet also cite complications such as skin bruising, neurovascular injury, and deep vein thrombosis as the drawbacks^8,9^. Likewise, antifibrinolytic agents such as tranexamic acid has variable dosing strategies, route and timing of administration despite their positive results^10^. The other measure, which is computer-assisted surgery, adds to the time as well as the cost of the surgical procedure. Due to this limitation, it may not be widely available in all centres performing TKR^5^.

The reported incidence of blood transfusion and blood loss following primary TKR varies widely in the literature. A nationwide analysis involving 228,316 patients from 922 US community hospitals showed an average predicted probability of blood transfusion of 6.3%, with 95% of the hospitals have predicted probability between 0.37% and 55%^11^. Another study of 4769 patients who underwent primary TKR showed that 14.6% of them received allogenic blood transfusion with a mean blood loss of 2181.4ml (SD 931.1) and mean haemoglobin drop of 3.0 g/dL (SD 1.2)^12^. In the US, considerable variation in transfusion rates highlights the uncertainty regarding the role of blood transfusion. Furthermore, a study has suggested that its use is discretionary and likely influenced by factors such as surgeons’ preference and local institutional guidelines^11^. In Malaysia, Husain reported that blood loss and requirements for blood transfusion following TKR can be reduced with intra-articular tranexamic acid administration^13^.

Allogenic blood transfusion can be lifesaving in certain clinical situations. However, it is also associated with several risks which include transfusion related acute lung injury (TRALI), transmission of pathogens and immunomodulation^14^. There is also an association between allogenic blood transfusion with a higher risk of postoperative prosthetic joint infection, mortality, thromboembolic events, and prolonged hospital stay, which ultimately will lead to increased resource utilisation^15-18^. Even autologous blood transfusion carries some risk. This group of donors tends to be older with multiple medical comorbidities, which leads to a higher risk of complications as well as unrecognised bacteraemia during blood collection^19^. Fortunately, in recent years there is growing interest to minimise blood transfusion in elective TKR surgery^19,20^.

To minimise perioperative allogenic blood transfusion in patients undergoing TKR surgery, there is a need for identification and establishment of risk factors associated with increased blood loss which subsequently led to allogenic blood transfusion^21^. A lower value of pre-operative haemoglobin is by far the most significant risk factor associated with allogenic blood transfusion as identified in multiple studies^22-24^. A few studies have also concluded that male gender, tourniquet time, and duration of surgery as predictors for an increase in blood loss following TKR surgery^3,12^. However, these forementioned results were based on studies conducted abroad with different patient ethnicity, surgical technique, and hospital guidelines for transfusion. At present, there is no local study which accesses the risk factors for allogenic blood transfusion and perioperative blood loss in TKR surgery.

It is imperative to perform a local study as genetic variation between different population has significant medical implications. According to the World Health Organization report on the global prevalence of anaemia in 2011, South-East Asia (SEA) has among the lowest mean haemoglobin concentration with the highest incidence of anaemia across population groups^25^. In addition to that, Malaysia has recorded a dramatic increase in the incidence of overweight and obesity within the population. Based on 2011 data, 33.6% of the Malaysian population was found to be overweight, with 19.5% of the population falling into the category of obesity^26^. These numbers should be alarming because when compared to White Caucasians, South Asians are at higher risk of developing obesity related non-communicable diseases such as osteoarthritis, coronary heart disease, metabolic syndrome, and type 2 diabetes mellitus^27^^29^.

These facts combined, further prove the importance of a study to assess the risk factors based on our Malaysian population. The knowledge obtained from this study will hopefully be able to assist the surgeon in preparing their patient for surgery as well as improving outcomes following surgery.

## Materials and Methods

This was a retrospective cross-sectional study involving a review of medical records of patients undergoing TKR surgery in the orthopaedic department at Hospital Melaka. TKR surgery in this centre was performed by six different surgeons. This research was conducted with approval from National Medical Research Register (NMRR) and the Ethical Committee of USM. A total of 296 adult patients who underwent elective TKR surgery during the period of 1st January 2015 to 31st December 2019 were identified and included in this study. The sample size was calculated using a formula from an article published in the *Indian Journal of Psychological Medicine* titled 'How to Calculate Sample Size for Different Study Designs in Medical Research'^30^. The pilot study used as a reference for this calculation was "TKA: risk factors for transfusion on South Asian population" published in BMC musculoskeletal disorder^18,21,31^.

The inclusion criteria were defined as diagnosis of primary knee osteoarthritis, procedure of primary TKR surgery and procedure performed on unilateral knee. Meanwhile, patients with haematological disorders and diagnosis of secondary knee osteoarthritis were not included. Other than that, patients who underwent revision TKR surgery, bilateral procedures, multiple operative procedures within the same setting and primary complex TKR surgery were also excluded. For the purpose of this study, primary complex surgery is defined as TKR procedure which requires the use of long stem prosthesis, wedge and/or bone graft to reconstruct bony defects. The main reasons for additional and specialised implants are major bone loss, major deformity of the knee as well as laxity of the collateral ligaments.

Standing AP and lateral views of both knees were obtained in all cases for pre-operative planning and templating. The procedure was performed by qualified surgeons under supervision of a senior consultant utilising similar surgical steps and techniques. The knee joint was approached using a standard anterior midline incision followed by a medial parapatellar retinacular incision. The anteromedial capsule was elevated subperiosteally along with the deep medial collateral ligament to expose the medial side of knee joint. The patella was then elevated laterally with the knee joint in the extended position. The femur and tibia were then prepared using an oscillating bone saw as well as checked for both flexion and extension gaps. Patella resurfacing was not routinely done as part of primary TKR in this centre. Once ready, the femoral and tibial prosthesis were affixed using bone cement to its position with polyethylene placed in between. A closed vacuum drainage systems was used in all cases and placed within the knee joint. The knee joint capsule was repaired in a water-tight manner, followed by subcutaneous and skin closure. Post-operatively, compressive bandaging was applied over the operated limb, and wound inspection was performed on the 3rd postoperative day.

Patients on antiplatelets or anticoagulants need to withhold their medications for 7 and 3 days respectively prior to surgery. All procedures were performed under subarachnoid block, combined spinal epidural or general anaesthesia. Pneumatic tourniquet was inflated in all cases just prior to skin incision and deflated following skin closure. Postoperative full blood count was taken at 24 hours post-surgery and documented in the case note. Patients were mobilised at day 3 post-operatively with walking frame assistance after check radiographs and removal of drains. None of the patients received pre-operative haemopoietic stimulation drugs. Post-operatively, all of them were prescribed dabigatran 110mg or 150mg once daily for a total duration of six weeks as prophylaxis against venous thromboembolism.

The intra-operative decision for blood transfusion was made by the anaesthetic team based on ongoing bleeding as well as the clinical condition of the patient. Post-operatively, the decision for blood transfusion was made based on *"Handbook on Clinical Use of Blood"* by National Blood Centre, Ministry of Health, Malaysia^31^. The transfusion criteria are as follows; 10g/dL is no longer used as a transfusion trigger, the most hemodynamically stable patient does not require transfusion if Hb is > 9.0g/dL, transfusion is almost always required if Hb is <6.0g/dL, symptomatic anaemia patient should be transfused regardless of haemoglobin level, and finally elderly patient or those with underlying cardio-respiratory disorder requires transfusion if Hb is <8.0g/dL.

Data entry and analysis were performed using IBM SPSS version 24.0, 64-bit edition for Windows by IBM Corporation 1989, 2016 USA. Descriptive analysis will be conducted on all variables. The data were presented in frequency and percentage for categorical data, while mean and standard deviation were used for numerical data. The relationship between blood transfusion as well as blood loss with various predictive factors will be analysed by fitting a logistic regression model.

## Results

A total of 296 patients were recruited for this study. The data obtained were expressed as mean with standard deviation for numerical variables. The categorical variables were expressed as frequency (n) and percentage (%) as tabulated in Table I. The mean age of patients was 64.10 years old (SD=8.50). The mean value of pre-operative Hb in all patients was 12.97 g/dL (SD=1.38) meanwhile the mean preoperative Hb in patients requiring transfusion was 10.97g/dL (SD=1.90). Mean for post-operative Hb in all patients was 11.41g/dL (SD=1.45). The result of the data showed that there were more female (n=221, 74.7%) compared to male patients (n=75, 25.3%).

**Table I:** Socio-demographic characteristics of all patients.

Independent Variables	Mean (SD)	n (%)
Age (years)	64.10 (8.50)	
Gender		
Males		75 (25.3)
Females		221 (74.7)
Pre-op Hb (g/dL); n=296	12.97 (1.38)	
Post-op Hb (g/dL); n=296	11.41 (1.45)	
Pre-op Hb needing transfusion(g/dL); n=13	10.97 (1.90)	
Pre-op Hb without transfusion(g/dL);n=283	13.07 (1.28)	
ASA score*	2.22 (0.54)	
Difference Pre-op and Post-op Hb(g/dL)	1.56 (0.82)	
BMI		
30 and less		164
>30		132 (44.6)
Types of anaesthesia		
Sub-arachnoid block		104 (35.1)
Combined spinal epidural		98 (33.1)
General anaesthesia		94 (31.8)
Tourniquet time (min)		
<120 min		218 (73.6)
>120 min		78 (26.4)
Antiplatelet/anticoagulant		
Yes		62 (20.9)
No		234 (79.1)
Surgery		
Right TKR		158 (53.4)
Left TKR		138 (46.6)

*American Society of Anaesthesiology (ASA) Score

A total of 158 (53.4%) of patients had an operation done on the right knee, whereas 138 (46.6%) were operated on their left knee. Only 62 (20.9%) of the patients were on antiplatelet or anticoagulant medications prior to operation, 164 (55.4%) of the patients had a BMI of 30 or less, while the remaining 132 (44.6%) fell into the category of obesity with a BMI of more than 30. There were almost equal number of patients in the three types of anaesthesia during surgery which were subarachnoid block, combined spinal epidural and general anaesthesia. A total of 218 (73.6%) of patients had a tourniquet time of less than 120 minutes; the remaining 78 (26.4%) had a prolonged tourniquet time of more than 120 minutes.

Based on our study, only 13 out of 296 patients received blood transfusion with a prevalence of 4.39% (95% confidence interval 2.04, 6.74). We analyzed variables associated with blood transfusion using multiple regression analysis (Table II). Pre-operative haemoglobin value was found to be the only significant variable associated with blood transfusion [P<0.001; Odds ratio (OR)=0.35; 95% Confidence interval (CI) 0.22,0.54. Therefore, our study showed that patients with minimum pre-operative Hb of 12.97g/dl had 65% reduced odds of transfusion.

**Table II:** Variables associated with blood transfusion.

Independent Variables	Simple logistic regression			Multiple logistic regression		
	Crude ^b^	Crude OR (95% CI)	p-value	Adjusted ^b^	Adjusted OR (95% CI)	p-value^b^
Age (years)						
<60	0	1				
≥60	0.91	2.50 (0.54, 11.49)	0.241			
Gender						
Males	0	1				
Females	1.45	4.25 (0.54, 33.24)	0.168			
BMI						
30 and less	0	1				
>30	-1.03	0.36 (0.10, 1.33)	0.125			
Types of anaesthesia						
Sub-arachnoid block	0	1				
Combined spinal epidural	0.49	1.63 (0.45, 5.96)	0.460			
General anaesthesia	-0.19	0.82 (0.18, 3.78)	0.804			
Pre-op Hb	-1.06	0.35 (0.22, 0.54)	<0.001	-1.06	0.35 (0.22, 0.54)	<0.001
Antiplatelet/anticoagulant						
Yes	0	1				
No	0.39	1.48 (0.32, 6.86)	0.616			
Tourniquet time (min)						
<120 min	0					
>120 min	-0.70	1				
ASA Score	-0.80	0.45 (0.16, 1.30)	0.140			
Surgery						
Right TKR	0	1				
Left TKR	0.62	1.88 (0.60, 5.90)	0.277			

We also examined the association between related variables and the quantity of blood loss (Table III). Multiple logistic regression analysis showed a significant association between tourniquet time of >120 minutes with an Hb drop of >2g/dL [P=0.001]. Participants with tourniquet time >120 minutes had 2.67 times the odds to have blood loss >2g/dL compared to participants with tourniquet time of less or equal to 120 minutes (95% CI=1.54, 4.64).

**Table III:** Variables influencing blood loss (Hb >2g/dL).

**Independent Variables**	**Simple logistic regression**		**Multiple logistic regression**	
	**Crude b (95% CI)**	**p-value**	**Adjusted b (95% CI)**	**p-value^b^**
ASA Score	0.87 (0.54, 1.41)	0.578		
Pre-op Hb				
Low	1			
Normal	1.90 (0.99, 3.63)	0.052		
Gender				
Males	1			
Females	0.72 (0.41, 1.28)	0.263		
BMI				
30 and less	1			
>30	0.95 (0.57, 1.60)	0.859		
Age (years)				
<60	1			
60 and more	1.12 (0.64, 1.97)	0.669		
Types of anaesthesia				
Sub-arachnoid block	1			
Combined spinal epidural	0.80 (0.43, 1.50)	0.485		
General anaesthesia	0.94 (0.51, 1.75)	0.853		
Tourniquet time (min)				
<120 min	1		1	
>120 min	2.67 (1.54, 4.64)	0.001	2.67 (1.54, 4.64)	0.001

Notes – ^b^ Forward multiple linear regression was applied.

## Discussion

We believe that the prevalence of blood transfusion in primary TKR varies between different centres and study populations The 4.39% prevalence of blood transfusion in our study was lower in comparison to those reported in the literature which ranged between 8–18%^12,17,24,32^. The study conducted by Mufarrih *et al*, which looked at the South Asian population, reported a 25% prevalence of blood transfusion following primary TKR surgery^33^. Their higher prevalence of transfusion can be attributed to inclusion of simultaneous bilateral knee procedure into the final analysis, whereas bilateral knee procedures and revision procedures were excluded from our study.

Bilateral knee procedures have been proven as a significant risk factor contributing to allogenic blood transfusion in multiple studies^33-34^. Nichols *et al* in 2016 found that although patients who underwent bilateral knee procedures were generally younger with less comorbidities compared to unilateral or revision groups, the prevalence of transfusion was more than double in their bilateral knee group^34^. The fact that a higher transfusion rate was observed following simultaneous bilateral knee procedures could be attributed to extensive bone cuts and surgical dissection^35,36^. On the other hand, staged bilateral knee procedures with an adequate interval in between will allow sufficient haematopoiesis to restore haemoglobin levels after the index procedure^37^.

In our study, the low prevalence of allogenic blood transfusion can be attributed to strict adherence to the Handbook on Clinical Use of Blood by the National Blood Centre, Ministry of Health Malaysia^31^. Among the recommendations were 10g/dL was no longer used as a transfusion trigger and that most haemodynamically stable adult patient can tolerate Hb >9g/dL. Other than that, trigger level of Hb in elderly patients with underlying respiratory or cardiovascular disease was <8g/dL. This handbook stressed that clinical evaluation on an individual basis was important and symptomatic anaemia patients should be transfused regardless of Hb level.

Based on our multiple regression analysis, the pre-operative haemoglobin value was found to be the only significant variable associated with blood transfusion. This result was consistent with multiple other studies conducted in Caucasian and South Asian populations^23,24,32^. Given the magnitude of TKR surgery, this fact was not surprising since low preoperative haemoglobin will further drop post-operatively to reach a transfusion trigger. Sehat *et al* examined the amount of visible and hidden blood loss following TKR surgery. They found that the mean total calculated blood loss was 1474ml and a drop of post-operative haemoglobin by 3.0g/dL can be expected^38^. Based on our study, patients who received allogenic blood transfusion had a mean pre-operative haemoglobin of 10.97g/dL. Given that 3.0g/dL drop in haemoglobin can be expected post-operatively, the final value will be just below the transfusion trigger. This finding highlights the importance of optimising pre-operative haemoglobin levels, particularly in anaemic patients.

Our Malaysian handbook for the rational use of blood and blood products also emphasised the treatment of pre-existing anaemia. This includes evaluation for iron deficiency anaemia, which, if present, should be treated with three months of haematinics prior to elective surgery. This handbook also suggested that any blood thinning medications be stopped 7 days before surgery^31^. In our study, antiplatelet and anticoagulant medications were withheld for 7 and 3 days, respectively. Cuenca *et al*, in their study, concluded that pre-operative haematinics combined with a transfusion protocol were able to reduce the incidence of allogenic blood transfusion after TKR surgery^39^. Unfortunately, pre-operative haematinics supplementation or treatment were not a routine practice at our centre. Apart from pre-operative haemoglobin, our study did not find any other factors which contributed to allogenic blood transfusion. In contrast, different population studies have identified that low body mass index, longer operation time, higher ASA status and female gender have significant association with allogenic blood transfusion^24,33,40^.

Our study also looked at several factors which might contribute to increase in blood loss following TKR surgery. Based on our analysis, prolonged tourniquet time of more than 120 minutes was the only variable that significantly contributed to this. The group of patients with prolonged tourniquet time has 2.67 greater odds of losing >2g/dL haemoglobin compared to their counterparts. In our centre, the standard practice was to temporarily deflate the tourniquet for a minimum of 15 minutes once the tourniquet time had reached 120 minutes. This brief period following tourniquet deflation will cause the return of circulation to the surgical area, which possibly contributed to an increasing in blood loss, evident by a drop in post-operative haemoglobin. Our results concur with findings from a meta-analysis conducted by Zhang *et al* on the timing of tourniquet release in total knee arthroplasty. Their meta-analysis concluded that releasing torniquet prior to wound closure causes higher volume of total blood loss as well as longer operation time^41^. From our analysis, we did not identify any other significant factors which contributed to increase in blood loss.

There were several limitations identified from our study. Firstly, due to the retrospective nature of this study, some data were not available for comparison as we were only able to retrieve information from pre-existing medical records to be included in the final analysis. However, we were able to trace a large number of samples to be included, spanning a period of five years. Secondly, TKR surgery in our centre was performed by six different surgeons with different levels of skills and experience. This could have contributed to different outcomes in relation to bleeding and blood loss. Fortunately, this variance had been minimised by all six surgeons utilising similar surgical techniques and approaches for their TKR surgery. On the contrary, the strength of our study was the presence of transfusion protocol based on the national guidelines to assist in the decision for allogeneic blood transfusion.

## Conclusion

Our study showed that prevalence of blood transfusion following primary TKR surgery was relatively low compared to other reported studies. Low pre-operative haemoglobin had been identified as a significant risk factor for allogenic blood transfusion. The Malaysian CPG recommends anaemia correction with haematinics to be started as early as three months prior to surgery. This information emphasises the importance of screening and correcting pre-operative anaemia especially before elective procedures. Every attempt should be made by the operating surgeon to minimise tourniquet time to reduce blood loss. Additional insights to guide surgeons in their daily clinical practice could be gained from further multicentre prospective studies investigating the impact of transfusions on patients' final clinical outcomes.
